# Biogenic Synthesis of Iron Oxide Nanoparticles Using *Enterococcus faecalis*: Adsorption of Hexavalent Chromium from Aqueous Solution and In Vitro Cytotoxicity Analysis

**DOI:** 10.3390/nano11123290

**Published:** 2021-12-03

**Authors:** Melvin S. Samuel, Saptashwa Datta, Narendhar Chandrasekar, Ramachandran Balaji, Ethiraj Selvarajan, Srikanth Vuppala

**Affiliations:** 1 School of Environmental Science and Engineering, Indian Institute of Technology, Kharagpur 21302, West Bengal, India; melvinsamuel08@gmail.com; 2 Department of Genetic Engineering, School of Bioengineering, SRM Institute of Science and Technology, Kattankulathur, Chennai 603203, Tamil Nadu, India; saptashwada.abc@gmail.com; 3 Department of Nanoscience and Technology, Sri Ramakrishna Engineering College, Coimbatore 641022, Tamil Nadu, India; narendhar.nano@gmail.com; 4 Department of Chemical Engineering and Biotechnology, National Taipei University of Technology, Taipei 10608, Taiwan; balajiyashik@gmail.com; 5 Department of Civil and Environmental Engineering, Politecnico di Milano, Piazza Leonardo da Vinci, 3220133 Milan, Italy

**Keywords:** A549 cells, chromium (VI), *E. faecalis*_RMSN6, exopolysaccharide, Fe_3_O_4_

## Abstract

The biological synthesis of nanoparticles is emerging as a potential method for nanoparticle synthesis due to its non-toxicity and simplicity. In the present study, a bacterium resistant to heavy metals was isolated from a metal-contaminated site and we aimed to report the synthesis of Fe_3_O_4_ nanoparticles via co-precipitation using bacterial exopolysaccharides (EPS) derived from *Enterococcus faecalis*_RMSN6 strains. A three-variable Box–Behnken design was used for determining the optimal conditions of the Fe_3_O_4_ NPs synthesis process. The synthesized Fe_3_O_4_ NPs were thoroughly characterized through multiple analytical techniques such as XRD, UV-Visible spectroscopy, FTIR spectroscopy and finally SEM analysis to understand the surface morphology. Fe_3_O_4_ NPs were then probed for the Cr(VI) ion adsorption studies. The important parameters such as optimization of initial concentration of Cr(VI) ions, effects of contact time, pH of the solution and contact time on quantity of Cr(VI) adsorbed were studied in detail. The maximum adsorption capacity of the nanoparticles was found to be 98.03 mg/g. The nanoparticles could retain up to 73% of their efficiency of chromium removal for up to 5 cycles. Additionally, prepared Fe_3_O_4_ NPs in the concentration were subjected to cytotoxicity studies using an MTT assay. The investigations using Fe_3_O_4_ NPs displayed a substantial dose-dependent effect on the A594 cells. The research elucidates that the Fe_3_O_4_ NPs synthesized from EPS of *E. faecalis*_RMSN6 can be used for the removal of heavy metal contaminants from wastewater.

## 1. Introduction

Magnetic nanoparticles play a critical role in various fields such as areas of bio-nanotechnology, drug delivery and biosorption applications [1,2,3]. To date, only dextran-coupled iron oxide nanoparticles are permitted by the U.S. Food and Drug Administration (FDA) [4]. Iron oxide nanoparticles have applications in various fields such as tissue engineering, immunoassay technology, cell separation, drug delivery, and heavy metal removal [5,6,7,8]. In recent years, a lot of research has been carried out to classify different types of iron oxide nanoparticles, especially the conjugates such as magnet viz, maghemite (Fe_2_O_3_) or magnetite (Fe_3_O_4_), among which magnetite has been found to be highly biocompatible [9]. Magnetite is a magnetic iron oxide with an inverse cubic spinel structure along with oxygen, forming a closed FCC bundle where Fe cations occupy the intestinal tetrahedral and octahedral positions [10]. Magnetite has a widespread application in the field of biomedical viz. (a) cellular therapy, (b) tissue repair, (c) drug delivery, (d) magnetic resonance imaging, (d) hyperthermia, etc. [11].

Magnetic iron oxide nanoparticles can be synthesized using various physical, chemical and biological methods [12]. Physical methods include the usage of gas-phase deposition and electron-beam lithography [13,14,15]. However, these techniques suffer from the limitation of controlling the size of the particle. The chemical method includes sleet of Fe^2+^ and Fe^3+^ aqueous salt solution by adding a base and is more efficient, with excellent control over the size and shape of the nanoparticle [16]. The conventional method involves adding a base to an aqueous solution mixture of Fe^2+^ and Fe^3+^ chloride ions at a molar ratio of 1:2. An alternative approach to produce the magnetic nanoparticle is by bacterial mediated synthesis [17]. Various types of nanoparticles have been synthesized using bacterial mediated biosynthesis such as zinc oxide, magnesium oxide nanoparticles, iron oxide nanoparticles etc. This is effective mainly due to its low cost, control over magnetic property, control over size and increased biocompatibility. However, the major drawback is the long time required for synthesis when compared to the other conventional methods [18]. Papers have previously reported bacterial synthesis of iron oxide nanoparticle—based on the redox properties in *Mycobacterium paratuberculosis*, *Shewanella oneidensis* and *Geothrix fermentans*, which could reduce Fe^3+^ by redox compounds, to serve as an electron shuttle between the microbe and the insoluble iron substrate [19,20,21].

Heavy metals and synthetic organic dyes are the important raw materials predominantly used in large-scale production of various drugs, textile materials and leathers [22]. It is worth mentioning that 10–15% of heavy metals and dye molecules are exerted into the environment during the treatment process [23]. Hexavalent Cr(VI) and trivalent Cr(III) are the most stable and prevalent forms of chromium (oxidation states 2 to + 6). Cr(VI) chemistry is strongly influenced by the pH and concentration of the solution, and it usually exists in the anionic form as Cr_2_O_7_^2−^ (dichromate), HCrO_4_^−^(hydrogen chromate), or CrO_4_^2−^ (chromate), depending on pH and concentration. H_2_CrO_4_ is the dominating species at pH values below 1 (chromic acid) and around 2 in acidic media. Dichromate (Cr_2_O_7_^2−^) ions are the most common type of Cr(VI). Cr_2_O_7_^2−^ and HCrO_4_^−^ ions are in equilibrium between pH 2 and 6, and Cr_2_O_7_^2−^ and HCrO_4_ ions are prominent as chromate anion in alkaline conditions (pH > 8). The vital issues arising due to these pollutants are very low biodegradability and less chemical stability in environmental aquatic systems [24]. In such a way, chromium is one of the most available elements on the globe, and its undeniable presence in water bodies and inland has escalated beyond its sustainable limit in recent decades [25]. The National Institute of Occupational Safety and Health (NIOSH), Washington, D.C, USA has listed chromate compounds as one of the leading causes of occupational lung cancer, and the U.S. Environmental Protection Agency (EPA) and the International Agency for Research on Cancer (IARC) have classified chromium as a human carcinogen, making Cr(VI) one of 33 compounds currently listed as posing the greatest potential health threat in urban areas. The chromium is present in two different modes such as chromium Cr (III) and Cr(IV) [26,27]. When human beings are in contact with chromium, it often leads to severe skin reactions and cancer in some cases. The possible dangers of Cr(VI) exposure by intake of drinking water have recently received more attention. Several short-term in vivo experiments conclusively demonstrated that Cr(VI) genotoxic ‘s potential is dose-dependent, with indications of a substantial threshold effect due to extracellular detoxification (by reduction to Cr(III)) prior to absorption by peripheral organs and tissues [28]. The toxic effect of Cr(IV) is way higher than trivalent Cr(III) ions. Therefore, the Cr(IV) is actively categorized as a group ‘A’ carcinogen as it shows teratogenic, carcinogenic and mutagenic traits. The main sources of Cr(IV) that release toxins to aquatic systems are wood-stocking industries, battery fabrication, dye-based industries, leather tanning, electroplating factories and metal polishing industries [29,30]. Currently, industries have commenced discovering alternate ways for treating Cr(IV) comprising wastewaters but at present, available Cr(IV) treatments from wastewaters were found to display reduced competence at lower concentrations. Although conventional methods viz. chemical reduction, electrochemical, ion-exchange and dialysis methods are being used for removing Cr(IV) ions from wastewater, they are relatively extortionate, inefficacious at lower concentrations and also lead to environmental difficulties of waste disposal [31,32,33]. Recently, researchers established several variegate materials that are extensively utilized for the removal of heavy metals, such as activated carbon [34], carbon nanotubes [35], polymers [36], resins [37], graphene materials [38]. Amongst all the materials, the Fe-based magnetic nanomaterials are widely preferred due to their alluring properties such as high specific surface area, simple synthesis process and no formation of by-products as a secondary pollutant [39]. Many iron oxide composite nano and micromaterials have been designed over the years for the remediation of various pollutants including heavy metals [40]. Various adsorbent materials such as mussel-inspired adsorbents, magnetic nano-adsorbents, and magnetic hydrogels have been utilized for the removal of pollutants from wastewater [41,42,43,44,45].

Microbial exopolysaccharides (EPSs) are classified as biopolymers. The EPSs are secreted in the extracellular medium through the microbial cells, which creates a slime-like substance clinging to the surface of the cells [46,47]. These formed compounds might be either a soluble or insoluble polymer that are released outside the cells and later to the environment [48]. Therefore, the synthesis of EPS is very significant for the microbial cells as it plays an important role in their survival [49]. Cell defense, connection to solid surfaces, cell aggregation, and cell–cell interactions are among these roles. EPS may be formed by different classes of microorganisms such as microbes, cyanobacteria, fungi and yeasts, and microalgae [50]. This investigation emphasizes the capacity of bacterial EPSs to play as reducing and stabilizing compounds during the bio-mediated production of metal nanoparticles for multifunctional application (heavy metal removal and cytotoxicity studies). We used a new bacterium, *E. faecalis*_RMSN6: (i) to extract EPS from *E. faecalis*, (ii) synthesis of highly stable iron oxide nanoparticle (Fe_3_O_4_ NPs) by coating EPS on Fe_3_O_4_NPs (iii) Fe_3_O_4_ NPs was characterized using different types of analytical instruments, (iv) to evaluate synthesized Fe_3_O_4_ NPs as adsorbents to remove Cr(VI) metal ions from an aqueous solution, and in vitro toxicity analysis of synthesized Fe_3_O_4_ NPs using EPS.

## 2. Materials and Methods

### 2.1. Materials

The precursors and the reagents were obtained from Sigma-Aldrich, Mumbai, Maharashtra, India. All the chemicals involved in the research were utilized without any further purification. The MRS agar and MRS broth were bought from HiMedia Laboratories Pvt. Ltd., Mumbai, Maharashtra, India.

### 2.2. Isolation, Screening, and Identification of Enterococcus faecalis_RMSN6 and Biosynthesis of Fe_3_O_4_ Nanoparticle

Samples were isolated from a metal-contaminated site from Vellore, Tamil Nadu in India. The isolated strains were screened for resistance against metals using the agar diffusion method. Briefly, plates comprising 20 mL tris-minimal agar medium were supplemented with varying concentrations (0, 10, 25, 50 and 100 mg/L) of chromium, copper, cadmium and lead. One of the bacterial samples showed resistance to the heavy metals, and bacteria were isolated and grown in MRS broth at 37 °C for 48 h [50]. The 16s RNA sequence of the bacteria was then sequenced using Illumina sequencing. The neighbour-joining phylogenetic tree of 16s rRNA gene sequence of bacterial strain *Enterococcus faecalis*_RMSN6 and most closely related species are shown in Figure S1 and the assembled Sequence for *E. faecalis*_RMSN6 (Figure S2). Primer details and PCR protocol are shown in Table S1. The bacterial isolate *E. faecalis*_RMSN6 was inoculated into a 250 mL Erlenmeyer flask containing 100 mL MRS broth in sterile condition at pH 7 and followed by incubation at 37 °C for 2 days. After the incubation period, the culture was centrifuged at 8000 rpm for 10 min at 4 °C. To the cell-free supernatant made up of EPS, 0.1 M iron chloride tetrahydrate (FeCl_2_.4H_2_O) and 0.2 M iron chloride hexahydrate (FeCl_3_.6H_2_O) were added into the flask. The flasks were then incubated in dark at room temperature, resulting in a dark brown color indicating the formation of Fe_3_O_4_NPs. To find out the effect of temperature, pH and incubation time of the nanoparticle formation, a range of temperatures (22, 27, 32, 37 and 55 °C), pH (2.0–7.0) and incubation times (20–120 h) was chosen for optimizing the nanoparticle synthesis.

### 2.3. Box-Behnken Design (BBD)

The investigative setup was constructed using a Box–Behnken design (BBD) for the analysis [51,52,53]. This model is ideal for quadratic surface fitting and also for the optimization process. The BBD is typically utilized for analyzing the effect of 3 independent variables ranging viz. pH (X1) (2.0–7.0), temperature factor (X2) (22–55 °C) and finally, duration of incubation (X3) (20–120 h) concerning the yield of the nanoparticle. The design Expert software was used to analyze the obtained investigative data (version 9, stat-Ease, Inc., Minneapolis, MN, USA).

### 2.4. Material Characterization

The UV-Vis spectrophotometer supplied from Sulpeco, India was used to measuring the absorption of bio-synthesized Fe_3_O_4_ NPs. Similarly, the FT-IR spectra for Fe_3_O_4_ NPs were performed in IR affinity-1, Shimadzu, Japan to analyze the functional groups. The Phillips PW 1830 instrument (Sulpeco, India) equipped with CuKα = 1.54 Å (operating conditions: 40 kV and 30 mA) was probed for obtaining the XRD spectra of bio-synthesized Fe_3_O_4_ NPs. The surface roughness of the Fe_3_O_4_ NPs was analyzed using an AFM instrument supplied by Nano surf AG (Gräubernstrasse, Liestal, Switzerland), Switzerland. The surface morphology and chemical composition of Fe_3_O_4_ NPs were studied using scanning electron microscopy from Bruker (Billerica, MA, USA). The Nanoparticle Analyzer SZ-100, Horiba Scientific (Miyanohigashi, Kisshoin, Minami-ku Kyoto, Japan) is utilized to understand the particle size and zeta potential of Fe_3_O_4_ NPs.

### 2.5. Adsorption Investigation

The experiments for adsorption of Cr(IV) using bio-synthesized Fe_3_O_4_ nanoparticles were performed in batches. The vital experimental parameters such as the influence of pH (1–9), contact time (0–600 min), the dosage of adsorbent (concentration 0.5–2.0 g/L) were investigated. The 20 mL reaction flask with Cr(IV) ion solution at varying concentrations ranging from 10–125 mg/L was made using the standard addition method for the adsorption experiments. The pH value of the standard solution was optimized to pH 2 with the Fe_3_O_4_ nanoparticle concentration of 0.5 g/L. Furthermore, the Fe_3_O_4_ nanoparticles were filtered well and the concentrations of Cr(IV) were calibrated using the 1,5-diphenylcarbazide method. The *RMSE*: root means square error Equations (2) and (3).

(1) $$q_{e}=\frac{C_{o}-C_{f}}{M}\times V$$

(2) $$R_{2}=1-\frac{\sum_{n=1}^{n}{\left(Y_{pred,.i}-y_{exp.i}\right)}^{2}}{\sum_{n=1}^{n}{\left(Y_{pred,.i}-y_{exp.i}\right)}^{2}}$$

(3) $$RMSE=\frac{1}{n-1}\sqrt{\sum_{n=1}^{n}{\left(Y_{pred,.i}-y_{exp.i}\right)}^{2}}$$

in the equations, the *C_o_* and *C_f_* correspond to the initial and equilibrium concentration of Cr(IV). The V(L) refers to the volume of the solution. The m(g) is for the adsorbent weight and consequently, *q_e_* (mg/g) indicates the adsorption capacity of the adsorbent.

### 2.6. MTT Assay Using A549 Cell Lines

The Lung cell lines (A549) were cultured in Dulbecco’s altered eagle medium reinforced with 10% Fetal Bovine Serum and 1% penicillin. An MTT assay was performed using A549 cell lines with varying concentrations of Fe_3_O_4_ NPs in a range of 10–200 μg/L. The absorbance values of the 96-well plate was quantified using ELISA. All the experiments were performed in triplicates. The IC50 value of the nanoparticles was calculated using GraphPad Prism 9.1.0 software, San Diego, CA, USA [50].

### 2.7. Statistical Analysis

All statistical analysis was done using GraphPad Prism 9.1.0 software, San Diego, CA, USA.

## 3. Results and Discussion

### 3.1. Synthesis and Physical Characterizations of Bio-Synthesized Fe_3_O_4_ Nanoparticles

In our study, we report the synthesis of Fe_3_O_4_ NPs by means of a biological method using iron chloride solution mixed with the culture supernatant of *E. faecalis*_RMSN6. When the colorless solution in the synthesis turned darkish brown, it confirmed the formation of Fe_3_O_4_ NPs. The color shift from colorless to brown happened due to the surface plasmon resonance (SPR) property of the material. The biosynthesis of Fe_3_O_4_ NPs through *E. faecalis*_RMSN6 also combined the physical synthesis process. The important advantages of the microbial synthesis technique are high yield, low cost and superior reproducibility. B. subtilis was used to isolate the *Aspergillus niger* YESM 1 from the Fe_3_O_4_ NPs. Then, the ferric oxide solution was combined with the supernatant of *B. subtilis* [54]. Finally, the formation of Fe_3_O_4_ NPs was confirmed by the change in color. The brick color indicates the unreduced Fe_3_O_4,_ and the dark brown color refers to the Fe_3_O_4_ NPs [55].

**Figure 1:**
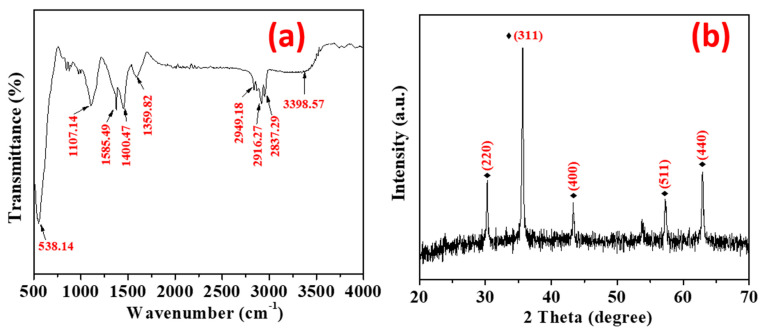
(**a**) FT-IR spectra of Fe_3_O_4_ NPs, (**b**) XRD patterns of Fe_3_O_4_ NPs.

The UV-vis spectrophotometry analysis confirmed the formation of Fe_3_O_4_ NPs, which gives rise to the surface plasmon resonance (SPR) adsorption band. The broad peak at 268 nm indicates the synthesis of Fe_3_O_4_ NPs. The adsorption peak is not stable; it can shift due to pH, incubation time, temperature, etc. The FTIR spectra for the biosynthesized Fe_3_O_4_ NPs are shown in Figure 1a. The peak at 2949, 2916 and 2837 cm^−1^ denote -H-C-H- and C=O stretching. The corresponding peaks at 1585, 1450 and 1359 cm^−1^ are assigned to amide groups and –CN- stretching amines; the peak at 1107 cm^−1^ is for the C-O stretch, and the peak arises due to the covalent linking of ester or ether groups to the nanoparticle. These results demonstrated that functional groups present in the EPS had successfully modified Fe_3_O_4_. The peaks at 875 cm^−1^ and 845 cm^−1^ are for the iron oxide. The X-ray diffraction pattern of the Fe_3_O_4_ NPs is shown in Figure 1b. The characteristic diffraction peaks arise at 2θ: 30.26, 35.56, 43.32, 53.79, 57.32 and 62.98. They are respectively belonging to (220), (311), (400), (422), (511) and (440) planes. These characteristic peaks are in agreement with standard diffraction data JCPDS: 39-1346 and correspond to face-centered cubic (fcc) symmetry [56].

**Figure 2:**
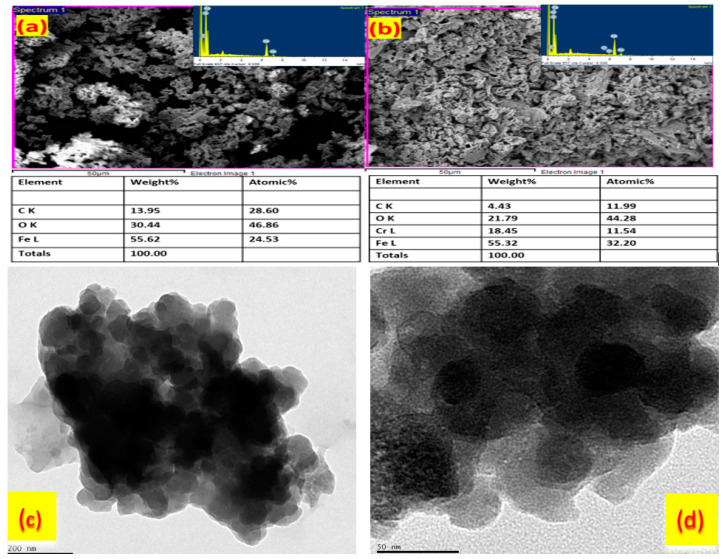
(**a**) SEM image shows the synthesized Fe_3_O_4_ NPs using B. amylolique faciens before Cr(VI) adsorption; (insert) EDX analysis of Fe_3_O_4_ NPs; (**b**) SEM analysis image of synthesized Fe_3_O_4_ NPs using B. amylolique faciens after Cr(VI) adsorption; EDX analysis of Fe_3_O_4_ NPs after Cr(VI) adsorption. TEM images of Fe_3_O_4_ nanoparticles showing the morphology of the nanoparticles at 200 nm (**c**) and 50 nm (**d**).

The SEM images confirm the presence of irregularly shaped particles (Figure 2). The particles can be seen to be coated in bio-molecular groups that lead to clumping of the nanoparticles (Figure 2). The SEM images of the Fe_3_O_4_ NPs were cubical, hexagonal, brick and irregular in shape (Figure 2a). The Figure 2a insert shows the EDX spectrum of Fe_3_O_4_ NPs synthesized using cell-free supernatant of *E. faecalis*_RMSN6; the Fe adsorption peak appears approximately at 0.7, 6.5 and 7 KeV due to surface plasmon resonance. In the Figure 2b insert, the EDX spectrum of Fe_3_O_4_ NPs after adsorption shows peaks approximately at 0.7, 6.5, 7.5 for Fe and 0.5, 5.3 and 5.9 for Cr(IV). Therefore, adsorption of Cr(IV) onto Fe_3_O_4_ NPs was confirmed by the EDX spectrum. The TEM images confirm the morphology of Fe_3_O_4_ nanoparticles, which is shown in Figure 2c,d. The Fe_3_O_4_ nanoparticles display a regular external surface, with wrinkles at center and smooth edges. Furthermore, using dynamic light scattering (DLS) analysis, the particle size of the Fe_3_O_4_ NPs was determined to be 15.4 nm. The Fe_3_O_4_ NPs stability was analyzed using zeta potential. The obtained zeta potential value was −29.6 mV. The value of zeta potential is directly proportional to the particle stability. The analysis showed that the stability of the synthesized iron oxide nanoparticle was higher.

BBD model for Fe_3_O_4_ NPs synthesis: The statistical analysis of BBD for Fe_3_O_4_ NPs yield by MSR5 strain exhibited a model F-value of 99.57, which implied the significance of the study. There was only a 0.01% chance that an F-value could have occurred due to noise. In addition, the values of “Prob > F” less than 0.0500 indicated that the model terms B, C, AB, A2, B, and C2 were significant.

The “Predicted R2” was estimated to be 0.878, and it is in proximity with “adjusted R2” 0.982. A ratio greater than 4 is desirable for “adequate precision”, and a ratio of 27.05 was obtained. The BBD model was used for navigating the design space.

**Figure 3:**
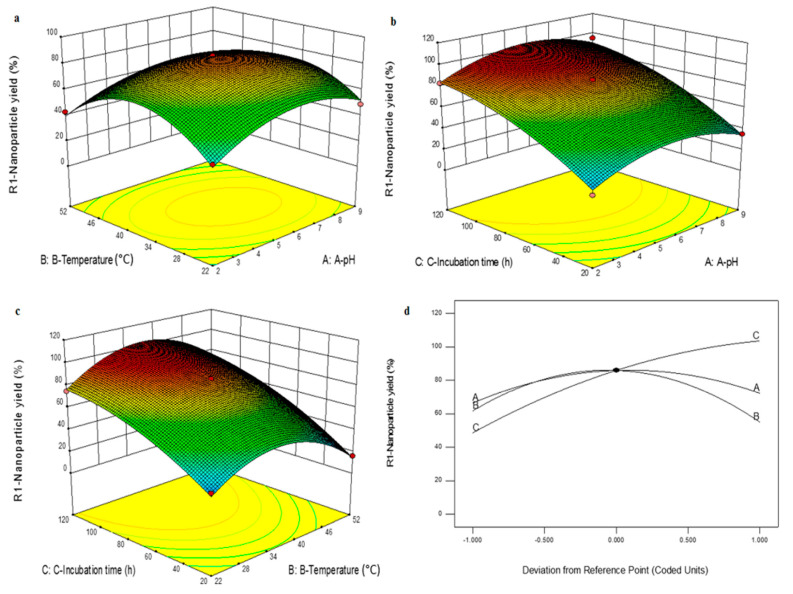
The 3D response surface plots showing the effects of interactions (**a**) effect of pH on Cr(VI) removal; (**b**) effect of temperature on removal of Cr(VI); (**c**) incubation time or contact time on Cr(VI) removal and (d) deviation from reference point (coded units). Note: A: effect of pH; B: effect of temperature; C: incubation time (min).

Figure 3 shows the effect of the variables viz. pH, temperature (°C) and incubation time (h), where maximum Fe_3_O_4_ NPs yield percentage (95%) was achieved at pH 5.5, temperature 37 °C and an incubation time of 70 h. The perturbation plot showed maximum Fe_3_O_4_ NPs yield at higher incubation time. With the increase in temperature, the decrease in the rate of formation of Fe_3_O_4_ NPs was witnessed. No formation of Fe_3_O_4_ NPs was observed when the temperature of a chemical reaction was higher than 45 °C. This was due to the fracture of biomolecules responsible for the reduction in Fe solution. Similarly, the rate of formation of Fe_3_O_4_ NPs was found to increase with the increase in pH. The increase in NP synthesis could be due to the formation of Fe (OH)_3_ in the initial part of the reaction, in the presence of Fe^3+^. When the pH was further increased, Fe (OH)_2_ was formed because of Fe^2+^ ions. As a result, Fe_3_O_4_NPs was found to be formed until the solution pH of 9.0 and then nucleation was found to occur. Likewise, an increase in the incubation time yielded a higher number of nanoparticles, with the highest yield at 120 h.

### 3.2. Effect of Various Parameters on Cr(VI) Removal

The effect of pH in the adsorption of Cr(IV) studies using Fe_3_O_4_ NPs was investigated. In this analysis, varying ranges of pH from 1 to 9 were selected and it was observed that Cr(IV) adsorption rapidly increased with the decrease in pH from 3. On the other hand, the adsorption capacity of Cr(IV) reduced with the increment in pH rising above pH 2. From the investigation, the ideal pH range was identified to be pH 2. The examination of the influence of adsorbent dosage during adsorption of Cr(IV) was conducted. With the increment in an adsorbent dose ranging from 0.25–1.0 g/L, the increase in removal rate of Cr(IV) using Fe_3_O_4_ NPs was witnessed. However, the adsorbent capacity of Cr(IV) using Fe_3_O_4_ NPs decreased from 93.802 mg/g to 40.83 mg/g when the dosage increased from 0.25 g/L to 1.0 g/L (Table S2).

**Figure 4:**
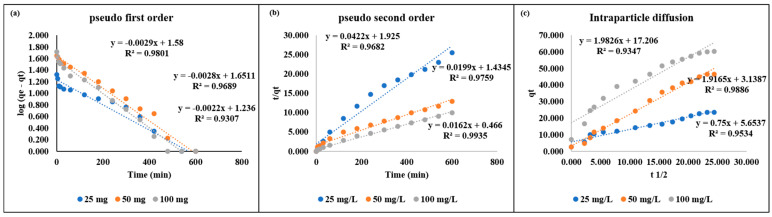
Kinetic modelling of the adsorption of Cr(VI) on Fe_3_O_4_NPs material, (**a**) pseudo-first-order kinetic plot, (**b**) pseudo-second-order kinetic plot, (**c**) intra-particle diffusion plot.

The influence of contact time for the adsorption of Cr(IV) using Fe_3_O_4_ NPs was briefly studied under 3 alternate Cr(IV) concentrations such as 25, 50 and 100 mg/L. The duration for this analysis was 0–600 min under the dose concentration of 0.5 g/L at 27 °C and agitation speed of 120 rpm. At the low concentration level of 50 mg/L, the superior Cr(IV) adsorption happened within 480 min (97% Cr(IV) removed). The quantity of Cr(IV) adsorbed using Fe_3_O_4_ NPs increased upon the increment in contact time. This is predominantly due to the presence of a large number of zones for adsorption and can be seen in Figure 4. Beyond the 480 min, the state of equilibrium was achieved for the adsorption capacity of Fe_3_O_4_ NPs.

### 3.3. Kinetic and Adsorption Isotherm Studies

**Figure 5:**
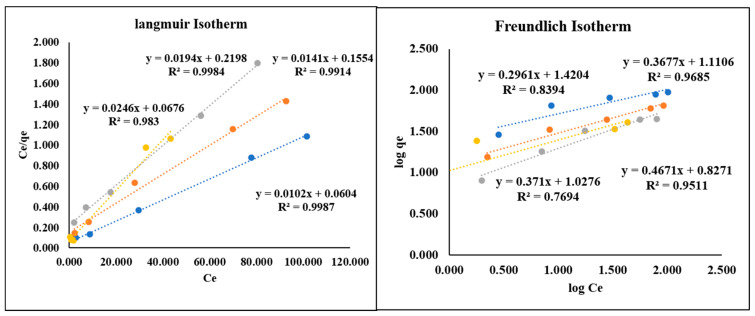
(**a**) Langmuir isotherm plot, (**b**) Freundlich isotherm plot for removal of Cr(VI) using Fe_3_O_4_ NPs material.

In the varying concentration of Cr(IV) from 25, 50 and 100 mg/L, the kinetic studies were carried out. The corresponding results are shown in Figure 5. The pseudo-first-order, pseudo-second-order and intra-particle diffusion models were adopted for the kinetic studies. The estimated outcomes are compiled in Table S3. The root means square error (*RMSE*) values are listed in Table S2. These values were used to evaluate the fitting of kinetic models. On comparing the 3 kinetic models, the pseudo-second-order model was identified to be desirable (Table S3). Using the Langmuir and Freundlich isotherm models, the adsorption isotherm for Cr(IV) was studied under different dose concentrations of 0.25–1.0 g/L (Figure 5). Upon the comparison, the Langmuir model was found to be an ideal fit, showing a monolayer surface adsorption. Obtained maximum capacity for the adsorption of Cr(IV) was found to be 98.03 mg/g and is presented in Table S4. This is significantly higher when compared to the recently reported literature. It is presented in Table S4 that pristine chitosan displayed 22.09 mg/g [57], the cyclodextrin-chitosan/GO had 61.31 mg/g [58], magnetic GO/chitosan composite showed 82.14 mg/g [59] and our biosynthesized Fe_3_O_4_ NPs showed enhanced adsorption 98.03 mg/g.

### 3.4. Adsorption Regeneration and Reuse

**Figure 6:**
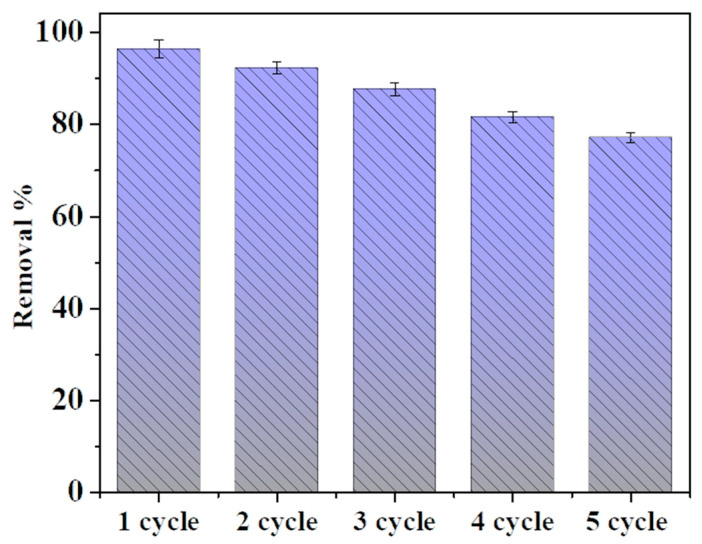
Regeneration study of Fe_3_O_4_NPs material.

The Cr(IV) desorption investigation using Fe_3_O_4_ NPs was performed using 1 mol/L NaOH conditions. The identical conditions were adopted for the successive adsorption investigation, and subsequently the adsorption capacity was examined. As an inference from studies, it was observed that the Fe_3_O_4_ NPs adsorbed Cr(IV) efficiently from the aquatic solution for the five cycles with a negligible decrease in adsorption capacity. The Fe_3_O_4_ nanostructures displayed an impressive 73% removal efficiency even after 5 cycles of investigation. From 92% for the first cycle, they decreased only slightly to 73% for the fifth cycle. They are shown in Figure 6. From previous studies it has been observed that there is a loss of adsorption capacity of iron oxide. These obtained results evidently indicate that the Fe_3_O_4_ NPs are extremely potent for the low-cost removal of Cr(IV) ions from the polluted water. There have been suggestions that over multiple reactions, the nanoparticles lose their stability and tend to aggregate, which leads them to lose their magnetic properties, hence causing reduction in reusability. Surface modifications help to increase the stability a great deal and also, as observed in our study modification by exo-polysaccharides, helped to increase the stability compared to many other studies conducted before. However further work is required to increase the stability of these nanoparticles even further, so as to strengthen them and increase their reusability [60,61,62,63,64].

### 3.5. Cytotoxicity Studies on A549 Cell Line

The cell viability and toxicity investigations were conducted on A549 cells. The MTT assay on A549 was performed using Fe_3_O_4_ NPs (cytotoxicity studies). As presented in Figure S3, the effect of Fe_3_O_4_ NPs under the varying concentrations of 10–200 μg/L on the A549 cell growth was investigated. Upon the increase in the concentration of Fe_3_O_4_ NPs, the % of cell death increased as well. The major alterations in the composition of cells were observed when effectively compared to control cells. The prepared Fe_3_O_4_ NPs inhibition concentration (IC_50_) was estimated to be 50 μg/L for the A549 cell lines. Therefore, it can be understood that the toxicity of Fe_3_O_4_ NPs depends on their size, form and charge.

## 4. Conclusions

The *E. faecalis*_RMSN6 showed the ability to synthesize iron oxide nanoparticles by extracellular supernatant. The *E. faecalis*_RMSN6 synthesized the metal nanoparticles extracellularly at a rapid rate. In such a way, we synthesized the Fe_3_O_4_ nanoparticles for the heavy metal removal and cytotoxicity analysis. The UV-Visible spectroscopy, FT-IR spectroscopy and XRD pattern vindicate the efficacy of *E. faecalis*_RMSN6 to synthesize Fe_3_O_4_ nanoparticles extracellularly. The size of Fe_3_O_4_ NPs was determined using SEM-EDX and DLS, and is in the range of 15–20 nm. The Fe_3_O_4_ NPs can potentially be utilized as alternate adsorbents to the traditional adsorbents for the efficient removal of heavy metal ions from polluted water.

This research investigation indicates that the adsorption equilibrium was achieved under 480 min. This shows the adsorption was mainly dependent on initial Cr(VI) concentration and pH. Ultimately, the highest level of Cr(VI) adsorption was observed at pH 2 under room temperature conditions, and adsorption capacity of the nanoparticles was found to be 98.03 mg/g. After 5 cycles of repeated experimentation, the nanoparticles retained up to 73% of their efficiency of chromium removal. The Langmuir isotherm model was the best fit compared to the Freundlich model. In vitro studies have shown synthesized Fe_3_O_4_ NPs to have elevated cytotoxicity activity against cell lines, suggesting the future therapeutic application of the nanoparticles. Overall, this analysis indicates the dual advantage of the biosynthesized Fe_3_O_4_ NPs, i.e., nanoparticles that could be used for the removal of chemicals or dyes from wastewater and possible use in biomedical applications.

## Data Availability

Not applicable.

## Supplementary Materials

The following are available online at https://www.mdpi.com/article/10.3390/nano11123290/s1. Figure S1: Neighbor- joining phylogenetic tree of 16s rRNA gene sequence of bacterial strain RMSN6 and most closely related species; Figure S2. Assembled Sequence for Sample RMSN6; Figure S3. Effect of biosynthesized Fe_3_O_4_NPs on A549 lung carcinoma epithelial cells at 50–250 μg/L for 24 h; Table S1. Primer Details; Table S2: Isotherm model regression constants for different experimental conditions; Table S3: Kinetics parameters for the adsorption of Cr(VI) by Fe_3_O_4_NPs material based on different concentrations; Table S4: Adsorption comparison of Fe_3_O_4_NPs and reported studies on adsorbents for Cr(VI); PCR Protocol; References [65,66,67,68,69,70,71,72,73,74] are cited in the supplementary materials..
