# Supplementary material for: Biogenic Synthesis of Iron Oxide Nanoparticles Using Enterococcus faecalis: Adsorption of Hexavalent Chromium from Aqueous Solution and In Vitro Cytotoxicity Analysis
Source: Nanomaterials (Basel). 2021 Dec 3;11(12):3290. doi: 10.3390/nano11123290 (PMC8705913; doi:10.3390/nano11123290)
Supplement: Supplementary file 1 [file nanomaterials-11-03290-s001.zip › nanomaterials-1470500-supplementary.pdf]

nanomaterials

Article

Biogenic Synthesis of Iron Oxide Nanoparticles using Enterococcus faecalis: Adsorption of Hexavalent Chromium from Aqueous Solution and in vitro Cytotoxicity Analysis

Melvin S. Samuel[1], Saptashwa Datta[2], Narendhar Chandrasekar[3], Ramachandran Balaji[4], Ethiraj Selvarajan[2*], Srikanth Vuppala[5*]

[1] School of Environmental Science and Engineering, Indian Institute of Technology, Kharagpur, West Bengal – 721 302, India; melvinsamuel08@gmail.com (M.S.S)
[2] Department of Genetic Engineering, School of Bioengineering, SRM Institute of Science and Technology, Kattankulathur, Chennai, Tamil Nadu, India; saptashwada.abc@gmail.com (S.D); selrajan@gmail.com (E.S)
[3] Department of Nanoscience and Technology, Sri Ramakrishna Engineering College, Coimbatore-641022, Tamil Nadu, India; narendhar.nano@gmail.com (N.C)
[4] Department of Chemical Engineering and Biotechnology, National Taipei University of Technology, Taipei, Taiwan, ROC; balajiyashik@gmail.com (R.B)
[5] Department of Civil and Environmental Engineering, Politecnico di Milano, Piazza Leonardo da Vinci, 32,20133 Milan, Italy; srikanth.vuppala@uniroma1.it (S.V)

* Correspondence: selrajan@gmail.com (E.T); srikanth.vuppala@uniroma1.it (S.V)

Sequencing protocol:

Single-pass sequencing was performed on each template using below 16s rRNA universal primers. The fluorescent-labeled fragments were purified from the unincorporated terminators with an ethanol precipitation protocol. The samples were resuspended in distilled water and subjected to electrophoresis in an ABI 3730xl sequencer (Applied Biosystems).

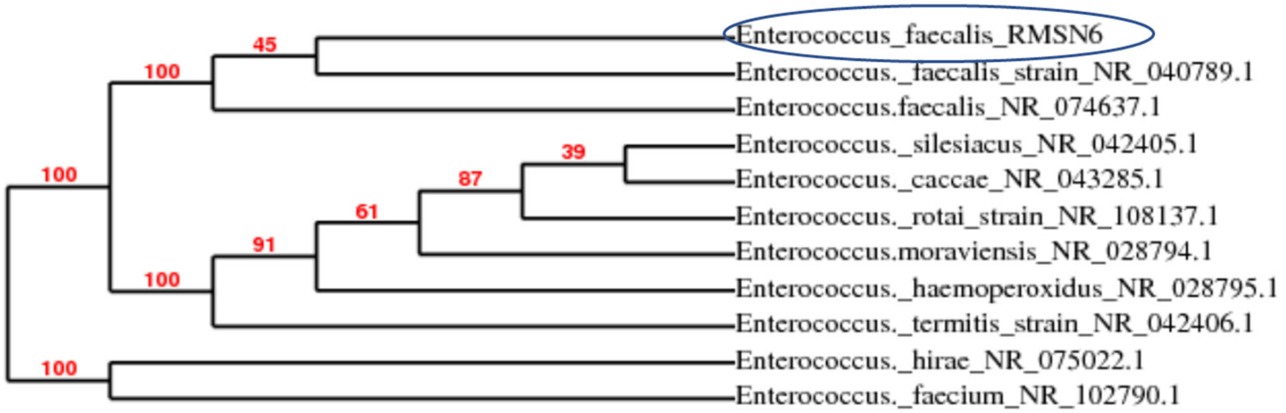

Figure S1: Neighbor- joining phylogenetic tree of 16s rRNA gene sequence of bacterial strain RMSN6 and most closely related species.

TTTGGGGGGGGTTTTTTTTGGGGTTTGTCCCGTCAGGACGAACGCTGGCGGCGTGCCTAATACATGCAAGTCGAACGCTTCTT
TCCTCCCGAGTGCTTGCATTCAATTGGAAAGAGGAGTGGCGGACGGGTGAGTAACACGTGGGTAACCTACCCATCAGAGGGG
GATAACACTTGGAAACAGGTGCTAATACCGCATAACAGTTTATGCCGCATGGCATAAGAGTGAAAGGCGCTTTCGGGTGTCGC
TGATGGATGGACCCGCGGTGCATTAGCTAGTTGGTGAGGTAACGGCTCACCAAGGCCACGATGCATAGCCGCCCTGAGAGGG
TGATCGGCCACACTGGGACTGAGCCACGGCCCAGATTCTTACGGGAGGCAGCAGTAGGGAATCTTCGGCAATGGACGAAAGT
CTGACCGAGCAACGCCGCGTGAGTGAAGAAGGTTTTCGGATCGTAAAATTCTGTTGTTAGAGAAGACCAAGGACGTTAGTAA
CTGAACGTCCCCTGACGGTATCTAACCAGAAAGCCACGGCTAACTACGTGCCAGCAGCCGCGGTAATACGTAGGTGGCAAGC
GTTGTCCGGATTTATTGGGCGTAAAGCGAGCGCAGGCGGTTTCTTAAGTCTGATGTGAAAGCCCCCGGCTCAACCGGGGAGG
GTCATTGGAAACTGGGAGACTTGAGTGCAGAAGAGGAGAGAAGGAATTCCATGTGTAGCGGTGAAATGCGTAGATATATGGA
GGAACACCAGTGGCGAAGGCGGCTCTCTGGTCTGTAACTGACGCTGAGGCTCGAAAGCGTGGGGAGCAAACAGGATTAGAT
ACCCTGGTAGTCCACGCCGTAAACGATGAGTGCTAAGTGTTGGAGGGTTTCCGCCCTTCAGTGCTGCAGCAAACGCATTAAGC
ACTCCGCCTGGGGAGTACGACCGCAAGGTTGAAACTCAAAGGAATTGACGGGGGCCCGCACAAGCGGTGGAGCATGTGGTT
TAATTCGAAGCAACGCGAAGAACCTTACCAGGTCTTGACATCCTTTGACCACTCTAGAGATAGAGCTTTCCCTTCGGGGACAA
AGTGACAGGTGGTGCATGGTTGTCGTCAGCTCGTGTCGTGAGATGTTGGGTTAAGTCCCGCAACGAGCGCAACCCTTATTGTT
AGTTGCCATCATTTAGTTGGGCACTCTAGCGAGACTGCCGGTGACAAACCGGAGGAAGGTGGGGATGACGTCAAATCATCAT
GCCCCTTATGACCTGGGCTACACACGTGCTACAATGGGAAGTACAACGAGTCGCTAGACCGCGAGGTCATGCTAATCTCTTAA
AGCTTCTCTCAGTTCGGATTGCAGGCTGCAACTCGCCTGCATGAAGCCGGAATCGCTAGTAATCGCGGATCAGCACGCCGCGG
TGGATAACGTTCCCGGGGCCTTGTACACACCGCCCGTCACACCACGAGAGTTTGTAACACCCGAAGTCGGTGAGGTAACCTT
TTTGGAGCCAGCCGCCTAAGGTGNGATAGATGATTGG

Figure S2. Assembled Sequence for Sample RMSN6

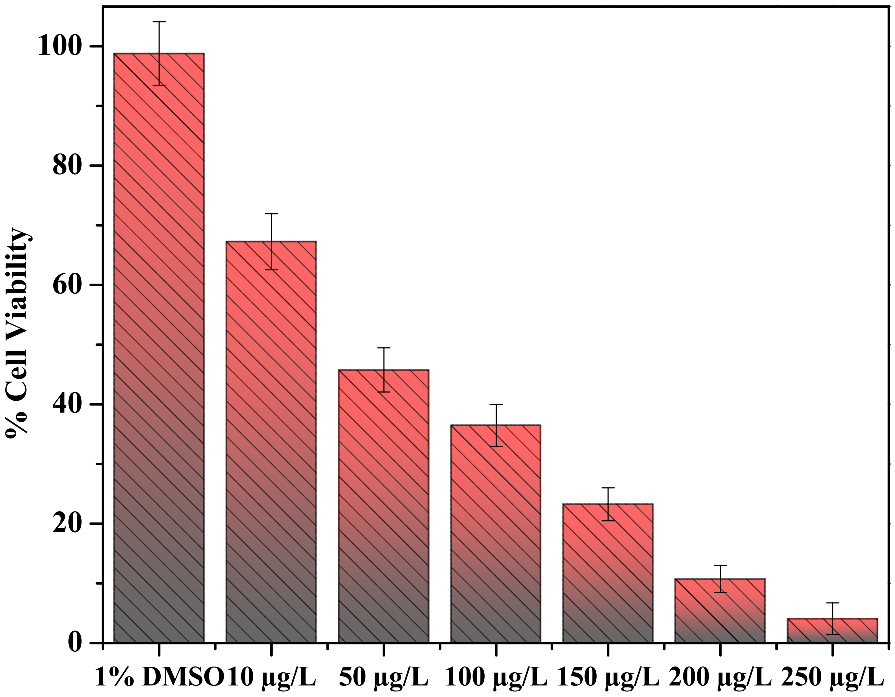

Figure S3. Effect of biosynthesized Fe_3O_4NPs on A549 lung carcinoma epithelial cells at 50-250 µg/L for 24h.

nanomaterials

MDPI

Table S1. Primer Details

Primer Details	Name Sequence	Number of Base
**27F**	AGAGTTTGATCMTGGCTCAG	20
**1492R**	TACGGYTACCTTGTTACGACTT	22

1. Bacterial Genomic DNA was isolated by using the InstaGeneTM Matrix Genomic DNA isolation kit Catalog # 732-6030
2. Using 16S rRNA Universal primers gene fragment was amplified using MJ Research PTC-225Peltier Thermal Cycler

PCR Protocol:

Add 1 μL of template DNA in 20 μL of PCR reaction solution. Use 27F/1492R primers for bacteria, and then perform 35 amplification cycles at 94 ºCfor 45 sec, 55 ºCfor 60 sec, and 72 ºC for 60 sec. DNA fragments are amplified about 1,400 bp in the case of bacteria. Include a positive control (E.coli genomic DNA) and a negative control in the PCR.

Purification of PCR products Removed unincorporated PCR primers and dNTPs from PCR products by using Montage PCR Clean up kit (Millipore).

The PCR product was sequenced using the 518F/800R primers. Sequencing reactions were performed using ABI PRISM® BigDyeTM Terminator Cycle Sequencing Kits with AmpliTaq®DNA polymerase (FS enzyme) (Applied Biosystems).

Table S2: Isotherm model regression constants for different experimental conditions

Isotherm models	Parameters	0.25 g/L	0.50 g/L	1.0 g/L	2.0 g/L
**Langmuir**	**q_{max}(mg/g)**	98.039	70.92	51.54	40.65
	**K_L (L/mg)**	0.168	0.09	0.088	0.0364
	**R^2**	0.9987	0.9914	0.9984	0.983
**Freundlich**	**K_f(mg/g)**	26.32	12.90	6.715	10.705
	**N**	3.377	2.719	2.140	2.695
	**R^2**	0.8394	0.9685	0.9511	0.7694

Table S3: Kinetics parameters for the adsorption of Cr(VI) by Fe_3O_4NPs material based on different concentrations.

**Concentration (mg/L)**	**25**	**50**	**100**
**Pseudo- first order**			
**K_1 (min^{-1})**	5.06 x 10^{-3}	6.44 x 10^{-3}	6.67 x 10^{-3}
**q_e(mg/g)**	17.218	44.781	38.018
**R^2**	0.9307	0.9689	0.9801
**RMSE**	0.546	1.125	1.045
**Pseudo– second order**			
**K_2 (min^{-1})**	0.021	0.0139	0.034
**q_e(mg/g)**	23.69	50.25	61.72
**R^2**	0.9682	0.9759	0.9935
**RMSE**	1.342	2.145	3.245
**Intraparticle diffusion**			
**R_{id}**	0.75	1.9165	1.982
**C**	5.6537	3.1387	17.206
**R^2**	0.9534	0.9886	0.9347

Table S4: Adsorption comparison of Fe_3O_4NPs and reported studies on adsorbents for Cr(VI).

**S. No.**	**Adsorbent material**	**q_{max} (mg/g)**	**Reference**
1	Ethylenediamine-modified cross-linked magnetic chitosan	51.813	[1]
2	Chitosan	22.09	[2]
3	Ceratocystis paradoxa MSR2	72.46	[3]
4	Chitosan cross-linked with epichlorohydrin	52.3	[4]
5	Magnetic chitosan nanoparticle	55.8	[5]
6	GO-CS@MOF [Zn(BDC)(DMF)]	144.92	[6]
7	$MnFe_2O_4$-chitosan	15.4	[7]
8	PANI/H-TNB composites	156.94	[8]
9	PVP/MoS_2	142.24	[9]
10	graphene oxide/chitosan/ferrite nanocomposite	270.27	[10]
11	chitosan grafted graphene oxide (CS-GO)	104.16	[11]
12	Magnetic porous carbonaceous (MPC)	21.23	[12]
13	GO-FH bio-nanocomposite material	212.76	[13]
14	***Extracellular Fe_3O_4NPs***	***98.03***	***This study***

References

1. Hu, X.-j.; Wang, J.-s.; Liu, Y.-g.; Li, X.; Zeng, G.-m.; Bao, Z.-l.; Zeng, X.-x.; Chen, A.-w.; Long, F. Adsorption of chromium (VI) by ethylenediamine-modified cross-linked magnetic chitosan resin: isotherms, kinetics and thermodynamics. Journal of hazardous materials 2011, 185, 306-314.
2. Aydın, Y.A.; Aksoy, N.D. Adsorption of chromium on chitosan: Optimization, kinetics and thermodynamics. Chemical Engineering Journal 2009, 151, 188-194.

3. Chidambaram, R. Isotherm modelling, kinetic study and optimization of batch parameters using response surface methodology for effective removal of Cr (VI) using fungal biomass. PloS one 2015, 10, e0116884.
4. Tianwei, T.; Xiaojing, H.; Weixia, D. Adsorption behaviour of metal ions on imprinted chitosan resin. Journal of Chemical Technology & Biotechnology: International Research in Process, Environmental & Clean Technology 2001, 76, 191-195.
5. Thinh, N.N.; Hanh, P.T.B.; Hoang, T.V.; Hoang, V.D.; Van Khoi, N.; Dai Lam, T. Magnetic chitosan nanoparticles for removal of Cr (VI) from aqueous solution. Materials Science and Engineering: C 2013, 33, 1214-1218.
6. Samuel, M.S.; Subramaniyan, V.; Bhattacharya, J.; Parthiban, C.; Chand, S.; Singh, N.P. A GO-CS@ MOF [Zn (BDC)(DMF)] material for the adsorption of chromium (VI) ions from aqueous solution. Composites Part B: Engineering 2018, 152, 116-125.
7. Badruddoza, A.Z.M.; Shawon, Z.B.Z.; Tay, W.J.D.; Hidajat, K.; Uddin, M.S. Fe3O4/cyclodextrin polymer nanocomposites for selective heavy metals removal from industrial wastewater. Carbohydrate polymers 2013, 91, 322-332.
8. Wen, T.; Fan, Q.; Tan, X.; Chen, Y.; Chen, C.; Xu, A.; Wang, X. A core–shell structure of polyaniline coated protonic titanate nanobelt composites for both Cr (VI) and humic acid removal. Polymer Chemistry 2016, 7, 785-794.
9. Wang, J.; Wang, X.; Zhao, G.; Song, G.; Chen, D.; Chen, H.; Xie, J.; Hayat, T.; Alsaedi, A.; Wang, X. Polyvinylpyrrolidone and polyacrylamide intercalated molybdenum disulfide as adsorbents for enhanced removal of chromium (VI) from aqueous solutions. Chemical Engineering Journal 2018, 334, 569-578.
10. Samuel, M.S.; Shah, S.S.; Subramaniyan, V.; Qureshi, T.; Bhattacharya, J.; Singh, N.P. Preparation of graphene oxide/chitosan/ferrite nanocomposite for Chromium (VI) removal from aqueous solution. International journal of biological macromolecules 2018, 119, 540-547.
11. Samuel, M.S.; Bhattacharya, J.; Raj, S.; Santhanam, N.; Singh, H.; Singh, N.P. Efficient removal of Chromium (VI) from aqueous solution using chitosan grafted graphene oxide (CS-GO) nanocomposite. International journal of biological macromolecules 2019, 121, 285-292.
12. Wen, T.; Wang, J.; Yu, S.; Chen, Z.; Hayat, T.; Wang, X. Magnetic porous carbonaceous material produced from tea waste for efficient removal of As (V), Cr (VI), humic acid, and dyes. ACS Sustainable Chemistry & Engineering 2017, 5, 4371-4380.
13. Samuel, M.S.; Subramaniyan, V.; Bhattacharya, J.; Chidambaram, R.; Qureshi, T.; Singh, N.P. Ultrasonic-assisted synthesis of graphene oxide–fungal hyphae: an efficient and reclaimable adsorbent for chromium (VI) removal from aqueous solution. Ultrasonics sonochemistry 2018, 48, 412-417.
